# Anti-Inflammatory Activity of *N*-(3-Florophenyl) ethylcaffeamide in Mice

**DOI:** 10.3390/ijms140815199

**Published:** 2013-07-24

**Authors:** Jung-Chun Liao, Jen-Chieh Tsai, Wen-Huang Peng, Yung-Jia Chiu, Ping-Jyun Sung, Minoru Tsuzoki, Yueh-Hsiung Kuo

**Affiliations:** 1 School of Pharmacy, College of Pharmacy, China Medical University, Taichung 404, Taiwan; E-Mail: ljc@mail.cmu.edu.tw; 2 Department of Health and Nutrition Biotechnology, College of Health Science, Asia University, Taichung 404, Taiwan; E-Mail: hope31269@yahoo.com.tw; 3 Department of Chinese Pharmaceutical Sciences and Chinese Medicine Resources, College of Pharmacy, China Medical University, Taichung 404, Taiwan; E-Mail: finsoul@gmail.com; 4 National Museum of Marine Biology and Aquarium, Pingtung 944, Taiwan; E-Mail: pjsung@nmmba.gov.tw; 5 Graduate Institute of Marine Biotechnology, National Dong Hwa University, Pingtung 944, Taiwan; 6 Nihon Pharmaceutica University, Saitama 362-0806, Japan; E-Mail: minoru-tsuzoki@nichiyaku.ac.jp; 7 Tsuzuki Institute for Traditional Medicine, China Medical University, Taichung 404, Taiwan

**Keywords:** anti-inflammatory, caffeamide, synthesis

## Abstract

In this study, we evaluated the anti-inflammatory activity of one synthetic product, *N*-(3-Florophenyl)ethylcaffeamide (abbrev. FECA), by using animal model of λ-carrageenan-induced paw edema in mice. The anti-inflammatory mechanism of FECA was determined by measuring the levels of cyclooxygenase-2 (COX-2), nitric oxide (NO), tumor necrosis factor (TNF-α), interleukin-1β (IL-1β), and malondialdehyde (MDA) in the edema paw tissue, and the activities of superoxide dismutase (SOD), glutathione peroxidase (GPx), and glutathione reductase (GRd) in the liver. The results showed that FECA reduced the paw edema at three, four and five hours after λ-carrageenan administration. The levels of COX-2, NO, TNF-α, and MDA in the λ-carrageenan-induced edema paws were reduced and the activities of SOD, GPx, and GRd in liver tissues were raised by FECA. These results suggested that FECA possessed anti-inflammatory activities and the anti-inflammatory mechanisms might be related to the decrease of the levels of COX-2, NO, and TNF-α in inflamed tissues and the increase in the MDA level by increasing the activities of SOD, GPx, and GRd.

## 1. Introduction

Inflammatory reaction, typically characterized by redness, swelling, heat, and pain, is one of the most important host defense mechanisms against invading pathogens. However, persistent or over-inflammation leads to tissue damage and possibly the failure of organs. Pro-inflammatory cytokines (e.g., TNF-α, IL-6, and IL-1β) are produced in large quantities by activated macrophages/monocytes that stimulate cellular responses via increasing prostaglandins (PGs) and reactive oxygen species (ROS). Additionally, lipid peroxidation (malondialdehyde, MDA) is produced by free radicals attacking the cell membranes. Thus, inflammatory effect results in the accumulation of MDA [1].

Many naturally occurring compounds, such as CAPE (caffeic acid phenethyl ester), resveratrol, quercetin, theaflavin, and curcumin are structurally similar to polyphenol, which is successfully employed in the prevention and treatment of a variety of diseases [2], including suppressing hepatic gluconeogenesis, stimulating glucose uptake, antihyperglycemia, and antiobesity effects [3–10]. CAPE is one of the major components of honeybee propolis and appears to exhibit antioxidant [11], anti-inflammatory [12], proapoptotic [13], antiviral [14], and immunomodulatory properties [15]. FECA is a derivative of CAPE. The anti-inflammatory effect of FECA is worthy of our continued investigation.

In this study, we investigated the anti-inflammatory activities of the FECA. Anti-inflammatory activity was determined by using λ-carrageenan induced mouse paw edema model. In order to evaluate the mechanism of anti-inflammatory effect, we also analyzed the levels of TNF-α, IL-1β, COX, MDA, and NO in the edema tissues, as well as the activities of superoxide dismutase (SOD), Glutathione peroxidase (GPx), and Glutathione reductase (GRd) in the liver.

## 2. Results

### 2.1. Effects of FECA on λ-Carrageenan-Induced Mice Paw Edema

**Figure 1:**
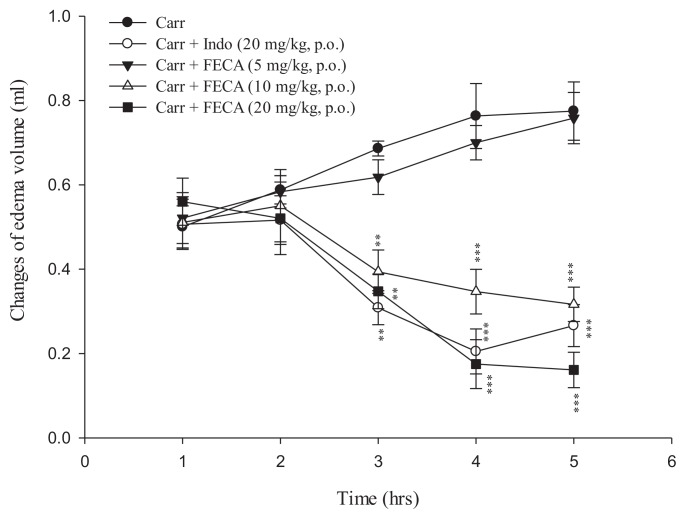
Effects of FECA and Indo on hind paw edema induced by λ-carrageenan in mice. Each value was represented as mean ± S.E.M. ** *p <* 0.01, *** *p <* 0.001 when compared to the λ-carrageenan (Carr.) group (one-way ANOVA followed by Scheffe’s multiple range test).

The results of λ-carrageenan-induced mice paw edema were shown in Figure 1, it was observed that *FECA* (10 and 20 mg/kg) and Indo (20 mg/kg) significantly inhibited the development of paw edema induced by carrageenan after three, four and five hours of treatment (*p* < 0.01–0.001).

### 2.2. Effects of FECA on COX-2 Level

**Figure 2:**
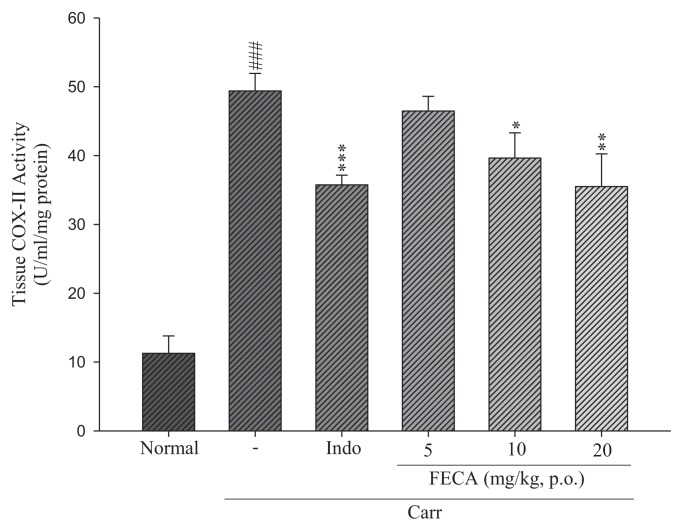
Effects of FECA and Indo on tissue COX-2 activity of edema paw in mice. Each value represents as mean ± S.E.M. ^###^*p* < 0.001 as compared with the normal group; * *p* < 0.05; ** *p* < 0.01; *** *p* < 0.001 as compared with the λ-carrageenan (Carr.) group (one-way ANOVA followed by Scheffe’s multiple range test).

The results showed that the activity of COX-2 increased significantly compared to the normal group in the edema paw of mice after carrageenan administration on the third hour (Figure 2). However, COX-2 activities were reduced significantly by treatments with FECA (10 and 20 mg/kg) and Indo (20 mg/kg) (*p* < 0.05–0.001).

### 2.3. Effects of FECA on TNF-α and IL-1β Levels

**Figure 3:**
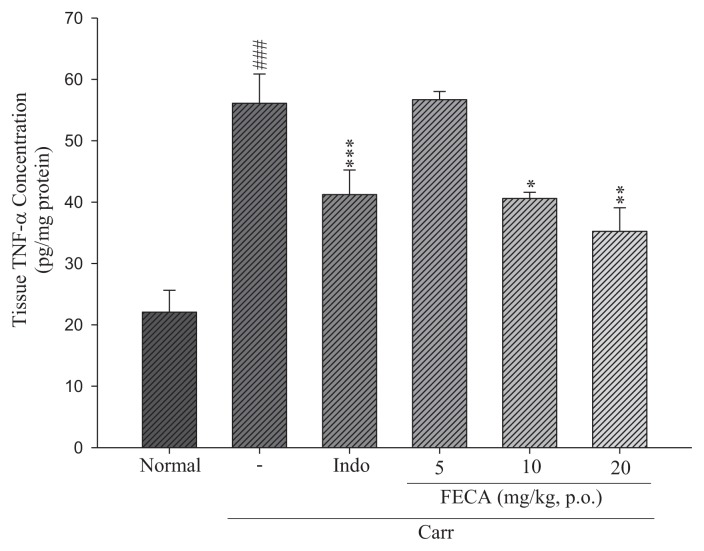
Effects of FECA and Indo on the tissue TNF-α concentration of edema paw in mice. Each value was represented as mean ± S.E.M. ^###^*p* < 0.001 as compared with the normal group; * *p* < 0.05; ** *p* < 0.01; *** *p* < 0.001 as compared to the λ-carrageenan (Carr.) group (one-way ANOVA followed by Scheffe’s multiple range test).

**Figure 4:**
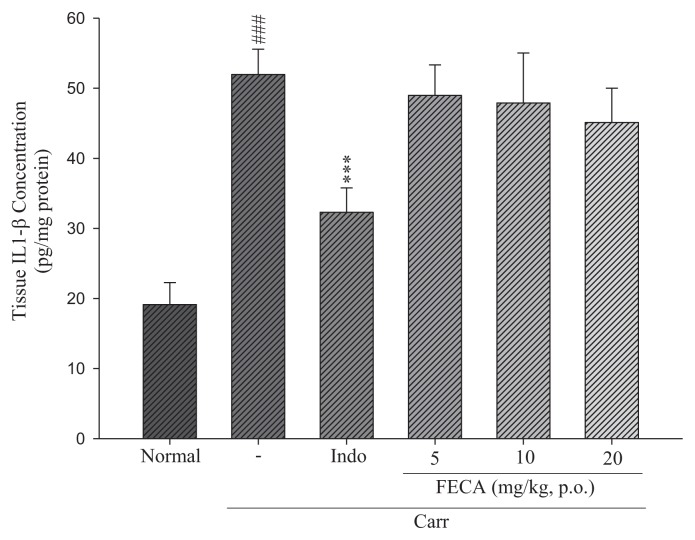
Effects of FECA and Indo on the tissue IL-1β concentration of edema paw in mice. Each value was represented as mean ± S.E.M. ^###^*p* < 0.001 as compared with the normal group; ** *p* < 0.01 as compared to the λ-carrageenan (Carr.) group (one-way ANOVA followed by Scheffe’s multiple range test).

The effects of FECA on TNF-α and IL-1β levels are shown in Figures 3 and 4. TNF-α and IL-1β levels in the λ-carrageenan induced edema paws were increased significantly compared to the normal group. The increased TNF-α levels were decreased by treatment with FECA (10 and 20 mg/kg) and Indo at 20 mg/kg (20 mg/kg) (*p* < 0.05–0.001, Figure 3). However, there are no significant changes on the IL-1β levels by treatment with FECA (Figure 4).

### 2.4. Effects of FECA on NO Level

**Figure 5:**
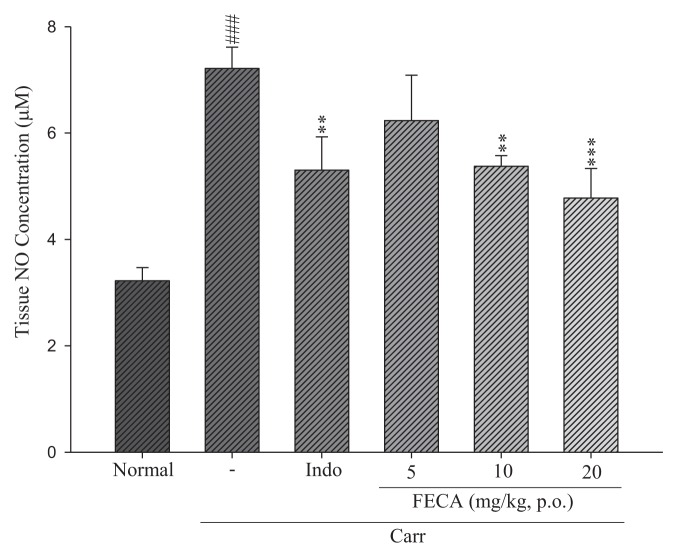
Effects of *FECA* and Indo on nitrate/nitrite concentration of edema paw in mice. Each value represents as mean ± S.E.M. ^###^*p* < 0.001 as compared with the normal group; ** *p* < 0.01; *** *p* < 0.001 as compared with the λ-carrageenan (Carr.) group (one-way ANOVA followed by Scheffe’s multiple range test).

As shown in Figure 5, the NO level in the edema paw induced by λ-carrageenan was significantly raised when compared with the normal group. There is a significant effect in the NO level when treating with FECA at 10 and 20 mg/kg, as well as Indo at 20 mg/kg (*p* < 0.01–0.001).

### 2.5. Effects of FECA on MDA Level Measurements

**Figure 6:**
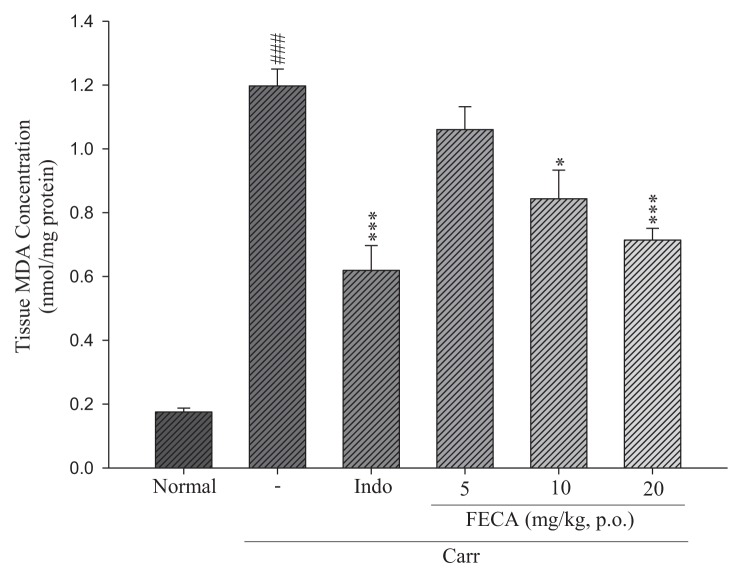
Effects of FECA and Indo on the tissue MDA concentration of edema paw in mice. Each value represents as mean ± S.E.M. ^###^*p* < 0.001 as compared with the normal group; * *p* < 0.05; *** *p* < 0.001 as compared with the λ-carrageenan (Carr.) group (one-way ANOVA followed by Scheffe’s multiple range test).

As shown in Figure 6, the levels of MDA in the edema paw induced by λ-carrageenan were significantly elevated when compared with the normal group. However, MDA levels were reduced by pretreatment with FECA 10 mg/kg (*p* < 0.05) and 20 mg/kg (*p* < 0.001), as well as Indo (20 mg/kg) (*p* < 0.001).

### 2.6. Measurements of Antioxidant Enzymatic Activities

The results of antioxidant enzymes such as SOD, GPx, and GRd at the 3rd hour following the intrapaw injection of λ*-*carrageenan in mice are presented in Table 1. SOD, GPx, and GRd activities in liver tissue were decreased significantly after λ-carrageenan administration at the 3rd hour, when compared with the normal group. Treatment with FECA at doses of 10 mg/kg and 20 mg/kg and Indo at a dose of 20 mg/kg increased the levels of SOD, and GRd activities significantly. In addition, the GPx levels were significantly raised by treatment with FECA at dose of 20 mg/kg.

## 3. Discussion

The anti-inflammatory activity of FECA was evaluated in λ-carrageenan-induced paw edema, a widely used model for screening the anti-edematous effect of various experimental compounds [16]. In the current study, FECA and Indo reduced the development of paw edema induced by carrageenan. These results illustrated that FECA had anti-inflammatory effect in λ-carrageenan-induced acute inflammation.

It is well known that λ-carrageenan-induced inflammation response immediately caused the release of several inflammatory mediators such as histamine, serotonin, and bradykinin, and then, further, the biosynthesis of prostaglandin (PG) and nitric oxide (NO) [17,18]. Inducible cyclooxygenase 2 (COX-2) is an inducible isoform and responsible for the biosynthesis of PG under inflammatory conditions [19]. Some inflammatory mediators, including TNF-α and IL-1β, are critical cytokines to the inflammatory responses and modulation of their production can improve therapeutic benefits [20]. TNF-α can cause immune responses by stimulating T cells and macrophages, and induce secretion of other inflammatory cytokines [21]. Previous investigations indicated significant correlations between cytokine production, COX-2 protein expression, and PG synthesis in the λ-carrageenan-induced paw tissues of mice [22]. In the present work, The COX-2 and TNF-α level in the edema paw tissues of mice were significantly diminished by treatment with FECA. Therefore, the mechanism of anti-inflammatory activity of FECA might act through the inhibitions of the COX-2 and TNF-α level in the model of λ-carrageenan-induced paw edema of mice. However, it seems no significant changes on the IL-1β levels by treatment with FECA occur. Whether FECA affects arachidonic acid release, it needs to be further studied in the future. TNF-α is produced primarily by mononuclear phagocytes and IL-1β is produced by a variety of cells. Both TNF-α and IL-1β are two important cytokines and can activate NF-κB pathway to cause inflammatory response [23]. In present study, FECA inhibited the TNF-α level but not IL-1β level. It is necessary to confer more thoroughly the reason that FECA inhibit TNF-α but not IL-1β in further studies.

NO is an important pro-inflammatory mediator produced by inducible nitric oxide synthase (iNOS) during conversion of l-arginine to l-citrulline [24]. The reaction of NO with superoxide anion will form a strong cytotoxic oxidant, peroxynitrite, which increases the production of PGs and causes lipid peroxidation and cellular damage, which [25]. Our results also confirmed that NO production was significantly raised in λ-carrageenan induced inflammation model. The increased level of NO was obviously reduced by treatments with FECA.

Current studies indicated that the λ-carrageenan-induced inflammation will cause the production of neutrophil-derived free radicals, such as hydrogen peroxide, superoxide, and hydroxyl radicals [26]. The λ-carrageenan-induced inflammation is used to research free radical generation in liver tissues after inflammatory states [27]. MDA formation is commonly used as a marker of free radical mediated lipid peroxidation injury and is thought to be due to free radicals attacking the plasma membrane [1,28]. Thus, inflammatory response would cause the accumulation of MDA. Our results indicated that the production of MDA was reduced by treatment of FECA. Treatment with FECA also significantly increased the SOD, GPx, and GRd activities. Thus, the results suggested that the suppression of MDA production may be due to the increases of SOD, GPx, and GRd activities. Another, the increase of SOD enhances the superoxide anion scavenging capacity and prevents the peroxynitrite-mediated tissue inflammatory response. Futhermore, the FECA’s structure is a derivative of CAPE. CAPE and its derivative exhibited free radical scavenger and antioxidant activity [29]. FECA may be a free radical scavenger/antioxidant compound.

## 4. Materials and Methods

### 4.1. Chemicals

**Scheme 1:**
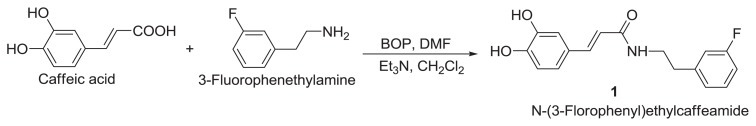
The synthetic procedure and chemical structure of FECA.

The following chemicals and reagents, λ-carrageenan, Indo, Griess reagent, *etc*., were purchased from Sigma-Aldrich Chemical Co. (St. Louis, MI, USA). The SOD, GPx, GRd, and MDA activity assay kits were purchased from Randox Laboratory Ltd. (Crumlin, UK). The NO and COX-2 assay kits were purchased from Cayman Chemicals Co. (Ann Arbor, MI, USA). Chemicals and enzyme immunometric assay kits for mouse IL-1β and TNF-α were obtained from eBioscience Inc. (San Diego, CA, USA). All of the other reagents used were analytical grade. FECA was obtained from the following method of amide binding coupling to prepare (Scheme 1). A solution of benzotriazol-1-yloxytris (dimethylamino) phosphonium hexafluorophosphate (BOP) (1.2 equiv) in dichloromethane (CH_2_Cl_2_) (5 mL) was added to a mixture of caffeic acid (100 mg), 3-fluorophenethylamine (1.2 equiv) and triethylamine (Et_3_N) (0.08 mL) in dimethylformamide (DMF) (1.0 mL). The mixture was stirred at 0 °C for 30 min, and then stirred at room temperature for 12 h. This reaction mixture was evaporated under vacuum, and the residue was partitioned between ethyl acetate (EtOAc) and H_2_O. Successively, the EtOAc layer was washed with 3 N aqueous HCl and 10% NaHCO_3_ (aq), dried over MgSO_4_, and concentrated in a vacuum. The residue was further purified by column chromatography with eluting solution (CH_2_Cl_2_-AcOEt 1:1, *v*/*v*) on silica gel (70–230 and 230–400 mesh, Merck 7734, Darmstadt, Germany). The final products (82% yield) were recrystallized from EtOAc to obtain pure crystals. ^1^H and ^13^C NMR spectra were recorded on a Bruker Avance 500 spectrometer (Bruker: Billerica, MA, USA). Electron impact mass spectra (EIMS) were determined on a Finnigan TSQ-46C mass spectrometer (Finnigan MAT, Waltham, CA, USA). IR spectra were recorded on a Nicolet Magna-IR 550 spectrophotometer (Nicolet, Madison, AL, USA).

The Indo and FECA were suspended in 0.5% CMC. The control animals were received 0.5% CMC (0.1 mL/10 g BW).

#### FECA

White solid; mp 186–188 °C. IR νmax (cm^−1^): 3436, 1652, 1619, 1520, 1440, 1358, 1115, 1015, 976, 852, 818. ^1^HNMR (CD_3_OD, 400 MHz): δ 4.47 (3H, s), 6.40 (1H, d, *J* = 15.6 Hz), 6.75 (1H, d, *J* = 8.0 Hz), 6.90 (1H, dd, *J* = 8.0, 2.0 Hz), 6.98 (1H, m), 7.01 (1H, d, *J* = 2.0 Hz), 7.03 (1H, m), 7.12 (1H, m), 7.33 (1H, m), 7.43 (1H, d, *J* = 15.6 Hz). EI-MS *m*/*z* (%): 109 (50), 124 (90), 163 (95), 247 (35), 287 (M^+^, 100).

### 4.2. Experimental Animals

Male ICR mice (20–25 g) were purchased from BioLASCO Taiwan Co., Ltd. (Ilan, Taiwan). The mice were raised in the animal center of China Medical University, at 22 ± 1 °C, relative humidity 55% ± 5%, with a light and dark cycle of 12 h for at least one week before the experiment. Animals were provided with a rodent diet and clean water *ad libitum*. Animal tests used in this study were conducted in accordance with the NIH Guide for the Care and Use of Laboratory Animals. All tests were conducted under the guidelines of the International Association for the Study of Pain [30]. The experimental protocol was approved by the Committee on Animal Research, China Medical University. Ether was used to anesthetize the animals before sacrificing them.

### 4.3. λ-Carrageenan-Induced Mice Paw Edema

The anti-inflammatory activities of FECA were determined by the λ-carrageenan-induced edema test in the hind paws of mice. Male ICR mice (10 per each group) were fasted for 24 h before the experiment with free access to water. Fifty microliters of a 1% λ-carrageenan suspension in saline was injected into the plantar side of right hind paws of the mice [31]. Paw volume was measured at 1, 2, 3, 4 and 5 h after the administration of the λ-carrageenan using a MK101 CMP plethysmometer (MuromachiKikai Co., Ltd., Tokyo, Japan). The degree of swelling was evaluated by the delta volume (*a – b*), where *a* and *b* were the volume of the right hind paw after and before the λ-carrageenan treatment, respectively. Indomethacin (20 mg/kg, *p.o.*) and FECA (5, 10 and 20 mg/kg, *p.o.*) were administered at 2 h after λ-carrageenan injection. The control group was given an equal volume of saline.

In the secondary experiment, the whole right hind paw tissues and liver tissues of the normal group (non-λ-carrageenan-induced) and the λ-carrageenan-induced groups were taken at the third hour. The right hind paw tissue was rinsed in ice-cold normal saline, and immediately placed in its four volumes of cold normal saline and homogenized at 4 °C. Then the homogenate was centrifuged at 12,000 rpm for 5 min. The supernatant was stored at −80 °C for the COX-2, NO, TNF-α, IL-1β, and MDA assays. Additionally, the whole liver tissue was rinsed in ice-cold normal saline, and immediately placed in an equal volume of cold normal saline and finally homogenized at 4 °C. Then the homogenate was centrifuged at 12,000 rpm for 5 min. The supernatant was obtained and stored at −80 °C for the antioxidant enzymes (SOD, GPx, and GRd) activity assays.

### 4.4. COX-2 Assay

COX-2 was measured by a quantitative sandwich enzyme immunoassay technique [32]. The capture antibody of COX-2 was seeded to each well of a 96-well plate overnight. The next day, a second set of biotinylated antibody was incubated with sample tissues or standard antigens in the plate before streptavidin-HRP was finally added. COX-2 was measured at 450 nm to determine their amount. The COX-2 activity was expressed as U/mL per protein (U/mL/mg protein).

### 4.5. NO Assay

NO was measured based on the method of Moshage [33]. For nitrite determination, nitrate was converted into nitrite utilizing nitrate reductase, NO_2_^−^ was measured by using the Griess reaction [34]. The absorbance of the final product (purplish red) was determined at 540 nm. Values obtained by this procedure represent the sum of nitrite and nitrate.

### 4.6. TNF-α and IL-1β Assays

TNF-α and IL-1β assays were measured by enzyme-linked immunosorbent assays (ELISA). Assays were performed according to manufacturer’s instructions. The amount of TNF-α and IL-1β were determined by reference to standard curves (0–1000 pg/mL) constructed in each assay. The concentrations of TNF-α and IL-1β in each sample were expressed as picogram per milligram protein (pg/mg) for cytokine concentration.

### 4.7. MDA Assay

MDA was evaluated by the thiobarbituric acid reacting substance (TBARS) method [35]. Briefly, MDA reacted with thiobarbituric acid in an acidic condition with high temperature (above 90 °C) and formed a red-complex TBARS. The absorbance of TBARS was determined at 532 nm.

### 4.8. Antioxidant Enzymatic Activity Measurements

The following biochemical parameters were analyzed to evaluate the antioxidant activities of FECA by the methods given below. SOD enzymatic activity was determined in accordance with the method of Misra and Fridovich [36] at room temperature. One hundred microliters of liver homogenate supernatant was added to 880 μL (0.05 M, pH 10.2, 0.1 mM EDTA) carbonate buffer. Twenty microliters of 30 mM epinephrine (in 0.05% acetic acid) was added to the mixture at 480 nm for 4 min on a Hitachi U 2000 Spectrophotometer. The enzymatic activity was expressed as the amount of enzyme that inhibits the oxidation of epinephrine by 50%, which is equal to one unit.

GPx enzyme activity was determined according to the method of Flohe and Günzler [37] at 37 °C. A reaction mixture consisted of 500 μL phosphate buffer, 100 μL 0.01 M GSH (reduced form), 100 μL 1.5 mM NADPH and 100 μL GRd (0.24 units). One hundred microliters of supernatant was added to the reaction mixture and incubated at 37 °C for 10 min. Then 50 μL of 12 mM *t*-butyl hydroperoxide was added to 450 μL of the tissue reaction mixture and measured at 340 nm for 180 s. The molar extinction coefficient of 6.22 × 10^−3^ was used to determine the enzymatic activity. One unit of activity was equal to the mM of NADPH oxidized/min/mg protein.

GRd enzyme activity was determined by the method of Carlberg and Mannervik [38] at 37 °C. Fifty microliters of NADPH (2 mM) in 10 mM Tris buffer (pH 7.0) was added in a cuvette containing 50 μL of GSSG (20 mM) in phosphate buffer. One hundred microliters of supernatant was added to the NADPH-GSSG buffered solution and measured at 340 nm for 3 min. The molar extinction coefficient of 6.22 × 10^−3^ was used to determine the GRd enzyme activity. One unit of activity was equal to the mM of NADPH oxidized/min/mg protein.

### 4.9. Statistical Analysis

All data were represented as mean ± S.E.M. Statistical analyses were performed with SPSS software. Statistical analyses were carried out using one-way ANOVA followed by Scheffe’s multiple range test.

## 5. Conclusions

In conclusion, these results indicated that FECA exhibited anti-inflammatory activities against λ-carrageenan-induced paw edema. The anti-inflammatory mechanism of FECA might be related to the inhibition of the formation of PGs by suppressing TNF-α, IL-1β and COX-2 levels and the reduction of MDA and NO productions by increasing the activities of SOD, GPx, and GRd activities. These findings supported that FECA may be developed into a pharmacological agent for the prevention or treatment of inflammatory diseases.

## Figures and Tables

**Table 1:** Effects of the FECA and Indo on the liver SOD, GPx, and GRd activities in mice.

Groups	SOD (U/mg protein)	GPx (U/mg protein)	GRd (U/mg protein)
Normal	61.70 ± 5.37	1.66 ± 0.12	0.114 ± 0.007
Carr.	44.15 ± 3.34 ##	1.17 ± 0.13 #	0.069 ± 0.004 ###
Carr. + Indo	58.48 ± 4.61 **	1.52 ± 0.03 *	0.099 ± 0.005 **
Carr. + FECA 5	47.61 ± 2.70	1.22 ± 0.06	0.073 ± 0.002
Carr. + FECA 10	54.46 ± 4.11 *	1.36 ± 0.11	0.089 ± 0.006 *
Carr. + FECA 20	57.62 ± 2.50 **	1.51 ± 0.07 *	0.097 ± 0.008 **

Each value represents as mean ± S.E.M.

# *p* < 0.05;

## *p* < 0.01;

### *p* < 0.001 as compared with the normal group.

* *p* < 0.05;

** *p* < 0.01 as compared with the Carr. (λ-carrageenan) group (one-way ANOVA followed by Scheffe’s multiple range test).
